# The dual role of alpha7 nicotinic acetylcholine receptor in inflammation-associated gastrointestinal cancers

**DOI:** 10.1016/j.heliyon.2020.e03611

**Published:** 2020-03-20

**Authors:** Khalil Hajiasgharzadeh, Mohammad Hossein Somi, Saeed Sadigh-Eteghad, Ahad Mokhtarzadeh, Dariush Shanehbandi, Behzad Mansoori, Ali Mohammadi, Mohammad Amin Doustvandi, Behzad Baradaran

**Affiliations:** a Immunology Research Center, Tabriz University of Medical Sciences, Tabriz, Iran; b Liver and Gastrointestinal Diseases Research Center, Tabriz University of Medical Sciences, Tabriz, Iran; c Neurosciences Research Center, Tabriz University of Medical Sciences, Tabriz, Iran; d Department of Cancer and Inflammation Research, Institute of Molecular Medicine, University of Southern Denmark, Odense, Denmark

**Keywords:** Clinical research, Gastrointestinal system, Immunology, Oncology, Pharmacology, Physiology, Alpha7 nicotinic acetylcholine receptor, Cholinergic anti-inflammatory pathway, Gastrointestinal cancers, Inflammation, Cancer regulation

## Abstract

Alpha7 nicotinic acetylcholine receptor (α7nAChR) is one of the main subtypes of nAChRs that modulates various cancer-related properties including proliferative, anti-apoptotic, pro-angiogenic and pro-metastatic activities in most of the cancers. It also plays a crucial role in inflammation control through the cholinergic anti-inflammatory pathway in numerous pathophysiological contexts. Such diverse physiological and pathological functions that initiate from this receptor may have significant impacts in determining the outcome of different cancers. Various tissues of gastrointestinal (GI) cancers such as gastric, colorectal, pancreatic and liver cancers have shown the up-regulated expression of α7nAChR as compared to normal adjacent tissues. According to the well-established connection between inflammation and tumorigenesis in the digestive system, there are mounting studies demonstrated either stimulatory or inhibitory effects of α7nAChR signaling in the development of GI cancers. To date, the precise underlying mechanisms related to this receptor in patients with GI cancers have not been fully elucidated. Regarding the paradoxical modulatory effects of this receptor in carcinogenesis, in this review, we aim to summarize the accumulated evidence about the involvement of α7nAChR in inflammation-associated GI cancers. It seems that the complex influences of α7nAChR may be a promising target in designing novel strategies in the treatment of such pathologic conditions.

## 1 Introduction

Gastrointestinal (GI) cancers is a common term that generally refers to the different types of cancers that influence the digestive system. These cancers originate from cells in the esophagus, stomach, exocrine pancreas, liver, gallbladder, biliary tract, small intestine, colon, rectum, and anus [1]. All of them are among the most frequent types of malignancies and represent an important public health problem with an immense burden on patients and societies worldwide [2]. Of note, gastric cancer, colorectal cancer and hepatocellular carcinoma (i.e. common type of liver cancer) are the preeminent causes of cancer-related deaths, so that they constitute ~25% of all cancer-related mortalities [2]. GI cancers are classified among multifactorial diseases caused by many different reasons, such as chronic inflammation and infection, environmental carcinogenic risk factors, and genetic predisposition in individuals [3].

In recent decades, mounting studies in humans, as well as several experimental studies in animals, points to crucial impacts of nicotinic acetylcholine receptors (nAChRs) in various cancers initiation, progression, and metastasis [4]. Among different subtypes of nAChRs, homo-pentameric alpha7-subtype of nAChR (α7nAChR) appears to be of particular significance in cancer research and is one of the major regulators of a wide variety of human cancers [5]. These receptors are named based on their activation by acetylcholine (ACh) as their predominant endogenous ligand and nicotine, which is an exogenous substance. They are functionally expressed by a variety of human cancer cell and tissues such as GI cancers. Many different GI tumor cells such as gastric [6], colorectal [7], pancreas [8], and liver cell types [9], as well as GI tumor-infiltrating immune cells including lymphocytes [10], monocytes [11], and macrophages [12] express α7nAChR subunits.

**Figure 1:**
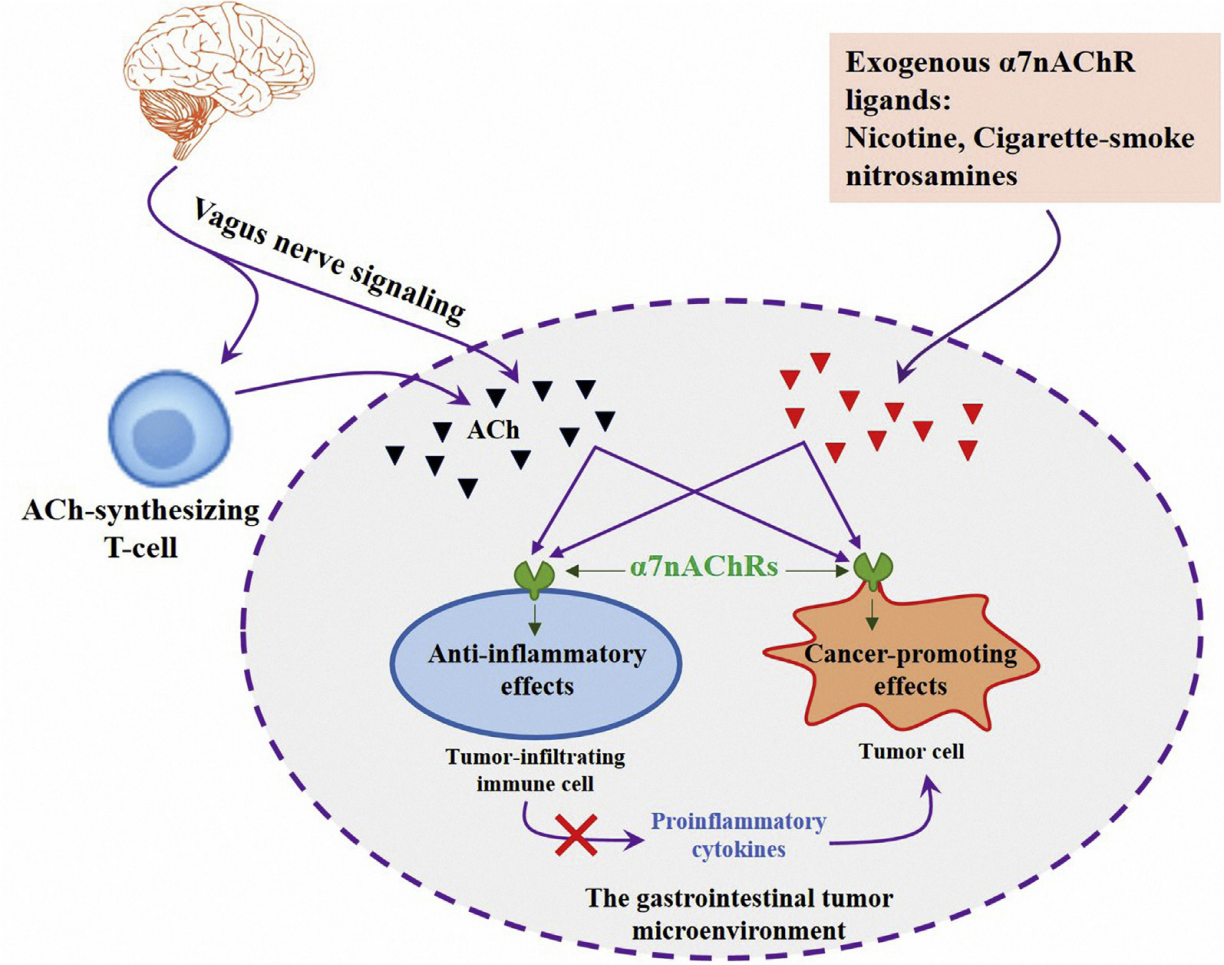
Schematic representation of the cancer-promoting and anti-inflammatory effects of α7nAChR in the gastrointestinal (GI) tumor microenvironment. Both of the GI tumor cells, as well as the GI tumor-infiltrating immune cells, express α7nAChRs which activated by several endogenous or exogenous ligands. These receptors exert either stimulatory or inhibitory effects in the GI-tumor processes. Such paradoxical effects should be carefully considered in the designing of novel pharmacological or surgical therapeutic strategies for the patients suffering from GI cancers.

Here, we highlighted an important and of growing interest topic of the role of α7nAChR in digestive carcinogenesis. Accumulating evidence has shown that increased activity of this receptor has been associated with cancer cell growth, survival, proliferation, angiogenesis, and metastasis [13, 14, 15]. Also, it has been reported that α7nAChR can participate in the regulation of tumor stem cells, tumor microenvironment, and epithelial-mesenchymal transition, thus promoting the progress of tumors [15, 16]. As another side of the same coin, α7nAChR is an essential regulator of inflammation and its anti-inflammatory function emerged as a novel therapeutic approach for inflammation-based diseases in recent years [17, 18]. This dual role of α7nAChR in the modulation of GI inflammation and cancers is the main subject of the current review (Figure 1). It seems that the diverse physiological actions and associated disorders have made of α7nAChR a promising target for effective drug development in GI cancers prevention and treatment.

## 2 GI inflammation and cancers

About two hundred years ago, Rudolph Virchow noticed that inflammatory cells have existed in tumor tissues proposing that persistent inflammation performs a central role in carcinogenesis [19]. Since then it has been identified that approximately twenty-five percent of all cancers are associated with chronic inflammation and mounting evidence implies that this inflammatory condition is one of the main precursors of some cancers [19]. Among these cancers, GI tract cancers are well-known cancer types that frequently arise as a result of chronic inflammation [20]. The inflammatory microenvironment has been considered as an intrinsic niche for these tumors development and progression [21]. In recent years, the causal relationship between activation of some inflammatory transcriptional factors such as nuclear factor-kappaB (NF-κB) and production of pro-inflammatory cytokines such as tumor necrosis factor-alpha (TNF-α) with cancers pathogenesis has been a major focus [22, 23]. The inflammatory responses promote both the proliferation and survival of tumor cells and also enhance their angiogenesis and metastasis [24]. On another hand, epidemiological studies suggest that anti-inflammatory drugs reduce the incidence rate and mortality from GI cancers [25]. Therefore, it seems that inhibition of inflammatory transcription factors and downregulation of pro-inflammatory cytokines may be valuable anticancer drug candidates [26]. Considering these facts, it is important to mention that the inflammation surely plays a role in carcinogenesis, but it is not the only phenomenon, and in some cancers, it is not the most important; but here our main intention is the inflammation-associated GI cancers and modulatory effects of α7nAChR in these types of cancers.

There are numerous findings indicate that the activation of α7nAChR and downstream signaling pathways could significantly attenuate inflammatory responses and suggests that this receptor could be an effective therapeutic target for inflammatory diseases [18, 27]. In these studies, α7nAChR activation via vagus nerve stimulation or other ACh synthesizing cells (e.g. T cells) [28], as well as exogenous agonists application possesses beneficial anti-inflammatory properties [29]. For the initial time, Tracey and colleagues exhibit that the “cholinergic anti-inflammatory pathway” (CIP) modulates the immune system through mechanisms that act on α7nAChR expressed on immune cells [17]. In their proposed model, ACh released from vagal terminals or from other ACh synthesizing cells interacts with α7nAChRs and suppressed pro-inflammatory cytokine production and inflammation [30]. The molecular mechanisms and signaling pathways that lead to the anti-inflammatory effects following induction of α7nAChR and inflammation-suppressing effects of vagus nerve have been reviewed in several articles and are not the subject of the current review [30]. To date, different studies have been conducted to investigate the anti-inflammatory actions of CIP and α7nAChRs in a variety of tissues including the GI system [31]. The outcome of such experiments has indicated that activation of α7nAChR initiates intracellular signal transduction cascades that significantly downregulates the nuclear translocation of NF-κB and attenuates the production of inflammatory cytokines such as TNF-α in numerous pathological conditions [32, 33, 34, 35, 36]. Therefore the therapeutic potential of α7nAChR in the inflammatory microenvironment of tumors and the protective role of the vagal nerve activity in carcinogenesis should be carefully considered [37]. Further to this immunomodulatory effect of α7nAChR, previous studies have shown that this receptor is one of the key molecules that mediate GI cancers initiation, progression, metastasis, and therapy responses. Table 1 summarizes the modulatory effects of α7nAChR in GI cancers regulation that have been investigated in previous studies. In the following sections, through a large number of such findings, we have detailed the most notable experiments that aimed to identify the role of gene expression and activation or blocking of α7nAChR in the modulation of inflammatory and carcinogenic responses in different parts of the digestive system. In addition, we discuss the implication of this receptor in the interplay between the cholinergic neural pathway and immune-related activities that regulate different pathophysiological events during GI carcinogenesis.

**Table 1:** Summary of some experimental studies indicating the modulatory effects of alpha7 nicotinic acetylcholine receptor (α7nAChR) in gastrointestinal cancers regulation.

Cancer type	Function	Commentary	References
GC	Enhanced metastasis	Nicotine and its derived nitrosamine compound (i.e. NNK) through activation of α7nAChR-related signaling pathways significantly enhanced gastric cancer (GC) metastasis.	[56, 57]
GC	Induced proliferation	Exposure to nicotine and NNK via α7nAChR mechanisms significantly induced cell proliferation in the AGS cell line.	[58]
GC	Enhanced chemosensitivity	α7nAChR enhanced the sensitivity of cancerous cells to chemotherapeutic reagents including taxanes and ixabepilone.	[6, 59]
GC	Enhanced chemoresistance	Targeted delivery of siRNA against α7nAChR makes gastric tumor cells became more resistant to 5-FU treatment.	[73]
CRC	Induced proliferation	Nicotine promotes cell proliferation via α7nAChR in human colorectal cancer (CRC) cell line.	[7]
CRC	Enhanced migration	Nicotine and tobacco-specific carcinogen (NNK) enhanced CRC cells migration through α7nAChR-mediated mechanisms.	[42, 43]
CRC	Enhanced tumor growth	ACh itself via α7nAChR serves as an important autocrine/paracrine growth factor in the human colon adenocarcinoma cell line HT-29.	[44]
CRC	Suppressed tumorigenesis	Nicotine suppresses acute colitis and CRC associated with chronic colitis in mice an effect that attenuated by the antagonist of α7nAChR.	[49]
CRC	Suppressed metastasis	α7nAChR in tumor-associated macrophages inhibits CRC metastasis in both animal model and LoVo human CRC cell line.	[52, 53]
PC	Induced proliferation	Nicotine and its nitrosated carcinogenic derivatives, promote cell proliferation of pancreatic cancer (PC) through activation of α7nAChR.	[8]
PC	Induced CSCs renewal	Nicotine induces self-renewal of pancreatic cancer stem cells (CSCs) through α7nAChR dependent mechanisms.	[92]
PC	Enhanced metastasis	Nicotine and cigarette-smoke promote the metastasis of PC via α7nAChR downstream signaling cascades.	[93]
LC	Induced proliferation	Nicotine acts through α7nAChR to stimulate the cholangiocyte proliferation in a xenograft mice model of liver cancer (LC).	[78]
LC	Increased carcinogenesis	NNK through increased expression of α7nAChR caused hepatic damage and LC progression in an experimental animal model.	[79]
LC	Enhanced tumor growth	Nicotine-triggered α7nAChR activation promotes both in vitro and in vivo tumor growth of HCC cells.	[81]

## 3 α7nAChR and colorectal cancer

Colorectal cancer (CRC) is one of the most serious worldwide health problems. This cancer causes at least seven-hundred thousand deaths every year, which makes it one of the most deadly cancers in the world [38]. Treatment of CRC consists of surgery, chemotherapy, and radiotherapy, but due to the poor effectiveness of such available treatments, the need to develop novel strategies for this cancer therapy (such as gene therapy) is urgent [38, 39]. The participation of the α7nAChR in this cancer pathogenesis has been investigated in previous studies. Activation of this receptor by nicotine contributes to CRC cell proliferation and inhibition of apoptosis [7, 40]. In these experiments, co-pretreatment of nicotine with α7nAChR antagonist or reduced expression of this receptor by transfection of specific siRNA abolished nicotine-stimulated cell proliferation [7]. Thus, it is postulated that this receptor plays an important modulatory function in mediating the proliferative effect of nicotine on colon cancer cells. In addition to cancerous cells proliferation, blocking and struggle against the metastatic character of this cancer remain a serious obstacle, while the majority of patients that suffer from CRC eventually die due to the metastatic disease [41]. Related to these leading causes of death, it has been shown that nicotine and NNK through α7nAChR-related signaling could enhance colon cancer cell migration and metastasis [42, 43]. Logically, both of the α7nAChR antagonist and α7nAChR siRNA methods have used to block the induced migration of CRC cells and the attenuated results confirmed that α7nAChR played the important role in nicotine and NNK stimulated cell migration in CRC [42, 43]. Further to the nicotine and nitrosamines, Pettersson and coworkers have shown that ACh itself via the α7nAChR serves as an important autocrine/paracrine growth factor in the human colon adenocarcinoma cell line HT-29 that positively contributes to the large intestine cancer progression [44].

In opposition to these tumor stimulatory effects, it also elucidated that, blockade of α7nAChR may disrupt the homeostasis and negatively influence the normal physiologic functions such as intestine immunology. The host inflammatory responses that recruit distinct immune cells, cytokines, and other immune mediators have a consistent positive association with all steps of CRC, including initiation, promotion, progression, and metastasis [45]. This reciprocal interaction highlights the need for identification of intrinsic immunomodulatory mechanisms such as nicotinic anti-inflammatory pathways in the CRC microenvironment regulation. It has been proposed that vagal signaling downregulates GI inflammation, and the mechanism is thought to involve ACh binding to the α7nAChR, as a modulator of CIP output [46]. In an animal model of colitis which induced by a chemical reagent (i.e. dextran sulfate sodium), vagotomy resulted in the increased level of NF-κB activation and aggravates colitis in animals [47]. Also, some other investigations have shown in cancer subjects that high vagal activity, reported by heart rate variability (HRV) indexes correlates with advantageous prognosis in the patients and supports the beneficial role of vagal activity in tumor modulation [48]. In addition, it has been shown that nicotine suppresses acute colitis and colon cancer which associated with chronic colitis in mice [49]. In this study, a specific antagonist of α7nAChR attenuated the inhibitory influences of nicotine on acute colitis [49]. These types of studies highlight the impact of anti-inflammatory mediators such as vagal anti-inflammatory signaling that mediated by α7nAChR on the outcome of patients with CRC [50, 51]. In another experiment, α7nAChR in tumor-associated macrophages inhibits CRC metastasis through its downstream intracellular signaling pathways which suggests that the expression of this receptor could be a valuable prognostic marker in CRC [52]. Similarly, Xiang and colleagues indicated that α7nAChR inhibits cell invasion and metastasis of LoVo human CRC cells through downstream signaling pathways that initiated from this receptor [53]. It is important to mention that, for CRC, we have to make a clear distinction between inflammation-associated cancers in patients with inflammatory conditions, such as inflammatory bowel disease, and cancers developed following the adenoma-adenocarcinoma sequence, since the role of α7nAChR in these two types of cancers may be different. Therefore, the anti-inflammatory and anti-cancer effects of α7nAChR should be carefully considered in developing novel CRC therapeutic strategies.

## 4 α7nAChR and gastric cancer

Gastric cancer (GC) is one of the top-ranked causes of cancer-related deaths. According to the global cancer statistics, approximately 950,000 new GC cases were reported, and due to poor effectiveness of available treatments, nearly 750,000 deaths occurred in the world during 2012 [2]. The incidence rate of this cancer demonstrates a strong etiologic association with smoking [54]. This habit emerged as a serious worldwide health problem and identified as an established environmental risk factor for GC development [54]. Smoking Tobacco contains various components that many of them have carcinogenic properties. Among these carcinogenic constituents of tobacco smoke, nicotine and nicotine-derived nitrosamines including 4-(methylnitrosamino)-1-(3-pyridyl)-1-butanone (NNK), N-nitrosonornicotine (NNN) and N-nitrosdiethylamine (DEN), have a great binding affinity to α7nAChR and partly by binding to this receptor exert their carcinogenic functions [55]. These agents activate numerous cellular signaling pathways downstream of α7nAChR, resulting in the stimulation of cancer-related properties.

Related to this fact, Wang and colleagues demonstrated that NNK through activation of α7nAChR significantly enhanced human gastric adenocarcinoma cells (AGS) migration [56]. In this study to investigate the mechanism of NNK-enhanced migration and verify that this effect is specifically mediated by α7nAChR, the selective α7nAChR antagonist, methyllycaconitine, was used in GC cells. In addition to this antagonist, α7nAChR expression level by small interfering RNA (siRNA) technology was silenced [56]. The results determined that both α7nAChR antagonist and α7nAChR-siRNA inhibited NNK-induced migration [56]. Similarly, Lien et al. have shown that GC cells exposure to nicotine itself caused enhanced gastric cancer metastasis specifically by α7nAChR, an effect suppressed by α7nAChR-siRNA transfection [57]. In another experiment, both nicotine and NNK significantly induced cell proliferation in AGS cells that expressed α7nAChR [58]. Treatment of these cells with α7nAChR antagonist α-bungarotoxin blocked these proliferative effects [58]. In addition, the silencing of the α7 receptors expression level in AGS gastric tumor cells by siRNA enhances the sensitivity of these cancerous cells to taxanes treatment [59]. Similarly, α7nAChR reduced the sensitivity of GC cells to ixabepilone as another chemotherapeutic reagent [6]. Therefore, the α7nAChR expression level in patients with GC may be a good indicator of chemotherapy drugs sensitivity. Altogether, it can be concluded that α7nAChR is an important regulator of gastric tumor cells proliferation, metastasis, and chemoresistance.

These studies highlighted the unfavorable consequences of exogenous activators of α7nAChR in GC regulation. On another hand, vagus nerve activation and subsequently, ACh release from its terminals as major endogenous sources of ACh, by binding to α7nAChR may play an additional stimulatory role in GC tumorigenesis [60]. It is delineated that the vagus nerve activation is necessary processes for gastric carcinogenesis [61]. Zhao and colleagues have shown that the vagus denervation of the stomach suppresses gastric tumorigenesis of the denervated portion in a mouse model of GC [62]. In this study, surgical removing of the vagus nerve (i.e. vagotomy) or pharmacological inhibition of vagus nerve signaling markedly reduced GC tumor incidence and progression [62]. This finding suggests that vagal denervation via reducing α7nAChR activity might represent a feasible strategy for the control of gastric tumorigenesis.

In addition to exogenous (e.g. nicotine and NNK) or endogenous (e.g. ACh from the vagal terminal) activators of α7nAChR and their carcinogenic functions, the GC etiology is closely linked to inflammation. This cancer is one of the best examples of inflammation-associated cancers in humans that is characterized by a slow stepwise evolution from superficial gastritis (inflammation of the lining of the stomach) to the final malignancy [63]. This sequence provides an excellent opportunity for the early detection and prevention of the underlying events preceding the development of the neoplasm [64]. However, the molecular mechanisms that regulate the transition from one step to the subsequent step are not still completely explained. In this cancer, chronic infection with *Helicobacter pylori (H. pylori)* is the strongest risk factor and ~90% of new cases of non-cardia GC worldwide attributed to this infection [65]. Infection with these bacteria causes an inflammatory response that enhances the infiltration of immune cells into the gastric mucosa [66]. Several studies clearly demonstrated the positive association between inflammation and pathogenesis of GC [67]. Recently, by RNA sequencing of gastritis and tumor tissues, it was indicated that the gene expression patterns of gastric tumors were similar to the gene expression of gastritis, indicating that these changes in the expression of tumors were induced by inflammation-dependent mechanisms [68]. Therefore, understanding the role of underlying molecular mechanisms involved in the modulation of inflammatory responses in GC pathogenesis is of particular importance.

Despite some studies claiming that the reduced activity of α7nAChR may successfully block GC growth and progression; but at the same time, it might also block intrinsic mechanisms that are essential for inflammatory reaction control. Relating to this concept, in opposition to the benefits of vagal inhibition in GC progression, some others suggest that α7nAChR signaling as well as high vagal activity predicts better cancer prognosis and also determined the increased risk of cancer mortality after vagotomy [69, 70]. Fujita et al. have shown the enhancement of gastric carcinogenesis following vagotomy in an experimental animal model [71]. Similarly, promotion by vagotomy and increased incidence of GC has been shown in another experiment in rats [72]. As another evidence that serves the beneficial effects of α7nAChR in GC, Chen et al. have shown that α7nAChR enhanced the sensitivity of GC cells to 5-fluorouracil (5-FU) as a well-known chemotherapeutic drug [73]. In this study, when the expression of this receptor was knock-downed by a specific siRNA targeting α7nAChR in AGS cells, these cells became more resistant to 5-FU treatment compared with the negative control cells [73]. Therefore, in opposition to the detrimental effects of α7nAChR in GC development and therapy responses, due to its other beneficial biological effects, the clinical application of α7nAChR antagonists in GC treatment needs to be taken into serious consideration.

## 5 α7nAChR and liver cancer

Liver cancer (LC) is the second most common cancer in males, the seventh in females and the third largest cause of cancer-related deaths worldwide [74]. The primary LC is initiated in the liver cells and is divided into different types, among them, hepatocellular carcinoma (HCC) is the most frequent malignant liver tumor and accounts for around ninety percent of the primary LCs [75]. However, this cancer is one of the common causes of cancer-associated deaths, the existing therapeutic options are not fully effective and the major approach is preventing LC at high-risk patients [76]. In an important study, Sakata et al. have shown that in humans, the highest accumulation of α7nAChR observed in the liver and this data demonstrated the importance of α7 receptor-related functions in this organ [77]. Similarly, it has been reported that α7nAChR is one of the predominantly expressed nicotinic receptors in both LC cell lines and primary hepatoma cells, demonstrating that these receptors may play crucial roles in the regulation of LC development and progression [9]. This receptor is more highly expressed in LC cells compared with normal cells and serves as a predominant protein responsible for nicotine-mediated LC progression [78]. Previous studies have shown the involvement of α7nAChR in liver pathological processes such as promoting carcinogenesis pathways in the liver. Aizawa et al. have shown that NNK as a high-affinity ligand of the α7nAChR through increased expression and activation of this receptor caused to hepatic damage and HCC progression in an animal model [79]. Similarly, Martínez and colleagues study suggest that nicotine acts through α7nAChR to stimulate the cholangiocyte proliferation in both *in vitro* and in xenograft mice model of LC [78]. In this context, the anti-tumor activity of acetylcholinesterase in HCC cells can also be justified by considering the fact that ACh is an important endogenous activator of α7nAChR [80]. Also, Wan et al. revealed that nicotine-triggered α7nAChR activation through signaling pathways associated with this receptor and promotes both *in vitro* and *in vivo* tumor growth of HCC cells [81].

There is numerous other evidence in the literature that displays the cancer-promoting effects of α7nAChR, but on the other hand, there are also some controversial finding about its detrimental effects in the liver. The liver reciprocally communicates with the brain partly via the hepatic vagus branch (HVB) and α7nAChR represents a unique molecular link between this parasympathetic system and immune- and cancer-related activities in the liver [82]. It is well-known that inflammatory processes are key players in LC development and progression [83]. Thus understanding the role of intrinsic inflammation controlling processes is of significant importance. In some animal models of experimental liver injury, cholinergic fibers are known to increase in the damaged area [84]. Also, the positive correlation between vagal activity that can be measured by different parameters of HRV and liver diseases has been well established in previous studies [85, 86]. These interactions may display the importance of CIP that exerts its protective effects through α7nAChR-dependent mechanisms in life-threatening conditions such as LC pathogenesis. In a most relevant study to this hypothesis which carried out by Hiramoto and coworkers, the protective role of the HVB activity against liver metastasis was founded in an experimental model in mice [87]. In addition, numerous findings demonstrated other beneficial effects of vagal firing in liver damage progression that exerts its protective effects via α7nAChR based signaling pathways [88, 89].

## 6 α7nAChR and pancreatic cancer

Pancreatic cancer (PC) is another leading cause of cancer-related deaths in the world, especially in developed countries [8]. Previous works have already established that PC cells express high levels of the α7nAChR subunit [90]. Similar to other GI cancers, nicotine is a major risk factor for this cancer but the underlying mechanisms are inadequately explained [91]. It has been shown that nicotine and its nitrosated carcinogenic derivatives, promote cell proliferation of PC through α7nAChR-mediated mechanisms [8]. In addition, PC stem cells stimulate tumor growth and metastasis, and it has been shown that nicotine induces self-renewal of these stem cells through α7nAChR dependent mechanisms [92]. In this study chronic exposure to nicotine increased the protein expression of the α7 receptor thereby promoting the malignant potential of human PC cells [92]. Momi and colleagues determined that nicotine promotes metastasis of PC via α7nAChR stimulation and subsequent activation of its downstream signaling cascades [93]. Also, chronic nicotine exposure inhibits the therapeutic effects of chemotherapy medications on PC in both *in vitro* and *in vivo* experiments [94]. By considering these findings, antagonizing the α7nAChR emerged a new therapeutic strategy for combating tobacco-related cancers [95]. For example, it was shown that human secreted Ly-6/uPAR related protein-1 (SLURP-1), an endogenous peptide antagonist of α7nAChR, is reported to have antiproliferative outcomes and significantly suppressed the development and progression of cancer-related characteristics in several cancer cell lines [96, 97]. Throm et al. indicated that SLURP-1 is a potential anti-malignant molecule that serves as an important tumor suppressor in PC [97]. In this study, it has been shown that patients with higher expression of α7nAChR and its endogenous ligand SLURP-1 in resected pancreatic tumors had a better survival rate compared to those with a lower level of these proteins expression [97].

In addition, it has been shown that some inflammatory regulators such as NF-κB activation and inflammatory cytokines production have a positive stimulatory role in PC associated properties such as proliferation and metastasis, thus inhibited activity of these inflammatory pathways by α7nAChR-based pathways may represent a promising anti-cancer approach in PC therapy [98, 99]. Regarding the well-established anti-inflammatory functions of the nicotinic neural pathway, it has been determined that blocked activity of this pathway by vagotomy or pretreatment with pharmacological inhibitors of α7nAChR resulted in enhanced severity of experimental pancreatitis and subsequent carcinogenesis [100]. The promoting effects of vagotomy on pancreatic carcinogenesis in animal models were demonstrated in previous studies [101, 102]. Recently, Partecke et al. demonstrated that subdiaphragmatic vagotomy caused increased tumor growth and worsened survival rate in an animal PC model [103]. They concluded that these cancer-promoting effects of vagal denervation occur due to the removed anti-inflammatory processes and subsequently, overproduction of inflammatory cytokines such as TNF-α in pancreatic tissue [103]. In normal physiological conditions, this excessive inflammatory process is restricted by CIP and α7nAChR-based signaling pathway. It is important to note that unbalanced activity of this receptor following surgical or pharmacological blocking may cause undesired inflammation and cancer-promoting effects. Thus, the anti-inflammatory function of vagal signaling should carefully considered in some operative procedures especially in the upper GI tract that in which partial or complete transection of the vagus nerve fibers usually will occur during the surgery.

## 7 Conclusion

This review highlighted the paradoxical effects of α7nAChR-related signaling pathways on GI tract cancers development and progression. As described in the text, several effects that depended on the presence and correct function of α7nAChR occurred in the regulation of GI cancers. Accumulated evidence demonstrated the cancer-promoting influences of this receptor in GI cancers, but, some other studies demonstrated beneficial effects for them in both cancer and non-cancer conditions. This evidence represents the excellent future perspectives of α7nAChR-based strategies in cancer therapy. We consider the GI cancers as well-known inflammation-associated cancers and don't address just the damaging effects of α7nAChR signaling in GI carcinogenesis, but somewhat concentrate on the anti-inflammatory functions of this receptor. This receptor exerts anti-inflammatory effects of vagal signaling and may serve the possible beneficial influences on GI cancers. There is an interesting hypothesis that supports the idea that the vagus nerve targets different organs and the brain via this neural pathway become informed about the preclinical neoplasms and suppressed them [104]. In accordance with this hypothesis, it should be mentioned that in some other non-cancer experiments the therapeutic benefits of α7nAChR agonists were used to improve wound healing processes as well as in the treatment of neurodegenerative illnesses such as Parkinson's and Alzheimer's diseases [105]. However, the impacts of other regulatory mechanisms such as single nucleotide polymorphism and regulatory microRNAs affecting α7 functions throughout the body should be investigated in future studies [106, 107]. Further insights regarding the therapeutic potential of α7nAChR that implicated in pathological conditions are discussed in a recent review and are beyond the scope of this manuscript to mention all of them [108]. The pathogenesis of GI cancers are complex disorders and not mediated by a single factor. Regarding this fact, the α7nAChR inhibitors have not been widely used for cancer prevention and treatment because of their possible complications. Hence, successful treatment may require comprehensive strategies to modulate several, rather than just a single underlying factor. On another hand, it is important to investigate intracellular mechanisms by which α7nAChR regulates GI tumor cells properties. Altogether, α7nAChRs are expressed in a variety of tissues including GI and hepatic cells and there is evidence to suggest that activation of these receptors has a paradoxical effect on the progression of cancer in the GI tract. On one hand, it suppresses inflammation and makes cellular microenvironment less favorable for tumorigenesis and on the other hand, activation of these receptors in cancerous cells promotes cell proliferation and migration. Therefore, in contrast to the benefits of their blockade in cancer therapy, the unique anti-inflammatory properties of them should be carefully considered in the designing of novel pharmacological or surgical therapeutic strategies.

## Declarations

### Author contribution statement

All authors listed have significantly contributed to the development and the writing of this article.

### Funding statement

This work was supported by the National Institute for Medical Research Development (NIMAD), Iran (project no. 972536) and Tabriz University of Medical Sciences, Tabriz, Iran (project no. 59256).

### Competing interest statement

The authors declare no conflict of interest.

### Additional information

No additional information is available for this paper.
